# Association between Residences in U.S. Northern Latitudes and Rheumatoid Arthritis: A Spatial Analysis of the Nurses’ Health Study

**DOI:** 10.1289/ehp.0901861

**Published:** 2010-03-25

**Authors:** Verónica M. Vieira, Jaime E. Hart, Thomas F. Webster, Janice Weinberg, Robin Puett, Francine Laden, Karen H. Costenbader, Elizabeth W. Karlson

**Affiliations:** 1 Department of Environmental Health, Boston University School of Public Health, Boston, Massachusetts, USA; 2 Channing Laboratory, Department of Medicine, Brigham and Women’s Hospital and Harvard Medical School, Boston, Massachusetts, USA; 3 Department of Epidemiology, Harvard School of Public Health, Boston, Massachusetts, USA; 4 Department of Biostatistics, Boston University School of Public Health, Boston, Massachusetts, USA; 5 South Carolina Cancer Prevention and Control Program, University of South Carolina, Columbia, South Carolina, USA; 6 Department of Environmental Health Sciences and; 7 Department of Epidemiology and Biostatistics, Arnold School of Public Health, University of South Carolina, Columbia, South Carolina, USA; 8 Exposure, Epidemiology, and Risk Program, Department of Environmental Health, Harvard School of Public Health, Boston, Massachusetts, USA; 9 Division of Rheumatology, Immunology, and Allergy, Brigham and Women’s Hospital and Harvard Medical School, Boston, Massachusetts, USA

**Keywords:** disease mapping, generalized additive models, geographic information systems (GIS), prospective cohort study, rheumatoid arthritis

## Abstract

**Background:**

The etiology of rheumatoid arthritis (RA) remains largely unknown, although epidemiologic studies suggest genetic and environmental factors may play a role. Geographic variation in incident RA has been observed at the regional level.

**Objective:**

Spatial analyses are a useful tool for confirming existing exposure hypotheses or generating new ones. To further explore the association between location and RA risk, we analyzed individual-level data from U.S. women in the Nurses’ Health Study, a nationwide cohort study.

**Methods:**

Participants included 461 incident RA cases and 9,220 controls with geocoded addresses; participants were followed from 1988 to 2002. We examined spatial variation using addresses at baseline in 1988 and at the time of case diagnosis or the censoring of controls. Generalized additive models (GAMs) were used to predict a continuous risk surface by smoothing on longitude and latitude while adjusting for known risk factors. Permutation tests were conducted to evaluate the overall importance of location and to identify, within the entire study area, those locations of statistically significant risk.

**Results:**

A statistically significant area of increased RA risk was identified in the northeast United States (*p*-value = 0.034). Risk was generally higher at northern latitudes, and it increased slightly when we used the nurses’ 1988 locations compared with those at the time of diagnosis or censoring. Crude and adjusted models produced similar results.

**Conclusions:**

Spatial analyses suggest women living in higher latitudes may be at greater risk for RA. Further, RA risk may be greater for locations that occur earlier in residential histories. These results illustrate the usefulness of GAM methods in generating hypotheses for future investigation and supporting existing hypotheses.

Rheumatoid arthritis (RA) is a chronic autoimmune disease with unknown etiology, although epidemiologic studies suggest genetic and environmental factors may play a role. Research on other chronic autoimmune diseases including lupus erythematosus, dermatomyositis, polymyositis, and vasculitis has shown geographic associations with higher latitudes (Gatenby et al. 2009; Hengstman et al. 2000; Somers et al. 2007; Walsh and Gilchrist 2006). Geographic variation in incident RA has been observed at the regional level according to state of residence (Costenbader et al. 2008a). The findings suggested an increased risk of RA for women who lived in the midwestern and eastern United States compared with the west, and the association was stronger with residency at ages 15 and 30 years than at baseline in 1976. In their review, Alamanos et al. (2006) also showed that RA varies geographically in areas of the world, with southern European countries having lower median incidence rates than northern European countries and North America. Ramos-Remus et al. (2007) observed that the mean age of RA onset was much younger among Mexicans than among Canadians. In another study, Anaya et al. (2001) found that RA is rare in African populations.

To explore further the association between location and RA risk, we analyzed individual- level residential data from U.S. women who participated in the Nurses’ Health Study (NHS). This prospective cohort study provides information on personal covariates and participant mobility prior to RA onset. Residential histories are particularly useful when exposures of interest are time dependent. We conducted spatial analyses that considered time measured by calendar year and by year of diagnosis for cases or censoring for controls. Generalized additive models (GAMs), a type of statistical model that combines smoothing with the ability to analyze binary outcome data and adjust for covariates, provide a useful framework for examining point data (Hastie and Tibshirani 1990; Kelsall and Diggle 1998; Webster et al. 2006). Using individual-level information and location in a GAM, we calculated the crude and adjusted odds ratios for incident RA in the United States. This method has the advantage of controlling for spatial confounders and of allowing for hypothesis testing for the significance of location in the disease maps. The objectives of the present analyses are to examine geographic variation at the individual level and to identify potential exposure hypotheses for further investigation.

## Methods

### Study population

We investigated the association between residence and incident RA using data from the NHS, a long-term prospective cohort study of U.S. female nurses. In 1976, at the study’s inception, 121,700 nurses, 30–55 years old, who lived in 11 states (California, Connecticut, Florida, Massachusetts, Maryland, Michigan, New Jersey, New York, Ohio, Pennsylvania, and Texas) and who were recruited through the state licensing boards, completed a mailed self-administered questionnaire and provided informed consent (Hart et al. 2009; Oh et al. 2005; Stampfer et al. 2000). Every 2 years, participants are mailed follow-up questionnaires to update current residential addresses, health outcomes, and behavioral risk factors. The addresses for 103,341 nurses from the 1988–2002 questionnaire cycles were geocoded, which contributed 173,624 addresses and 762,511 questionnaire records. Although nurses’ addresses were concentrated in the 11 original study states at baseline in 1976, by the follow-up period in 2002, the women were located in all 50 states. The medical records of nurses were reviewed to identify those who self-reported a diagnosis of RA. In this study, we used the diagnostic criteria of the American College of Rheumatology for RA (Karlson et al. 1995, 2004). For 1988 through 2002, we identified a total of 461 women with confirmed incident RA. When the participants reported having RA, their information was censored, and their residential history was considered complete at that time. We used incidence density sampling to randomly select 20 controls per case from among the noncases for a total of 9,220 controls (Richardson 2004). Selecting 20 controls per case allowed us to analyze the geographic distribution of the population under study and to keep the numbers reasonable for computation. Women who were noncases with a geocoded address at the time a case was diagnosed were eligible to be a control. Once selected, information for controls was censored, and their residential history was considered complete at that time. Thus, the proportion of participants censored in each year was the same for cases and controls.

### Spatial analysis

We estimated disease odds using GAMs, a form of nonparametric or semiparametric regression with the ability to analyze binary and continuous outcome data while adjusting for covariates (Hastie and Tibshirani 1990; Kelsall and Diggle 1998; Webster et al. 2006). We modeled location, a potential surrogate measure of exposure, using a bivariate smooth (S) of longitude and latitude (x_1_) and (x_2_)


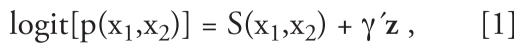


where the left-hand side is the log of the disease odds at location (x_1_,x_2_), **z** is a vector of covariates, and γ is a vector of parameters. The model is semiparametric because it has the nonparametric smooth, but the covariates are modeled parametrically. Without the smooth function, S(x_1_,x_2_), the model becomes an ordinary logistic regression on the covariates. To examine if timing of residential location impacts RA risk, we conducted analyses using 1988 addresses (the earliest available that were geocoded) and the addresses at time of diagnosis or censoring for all participants.

Spatial confounding occurs when risk factors for a disease are not evenly distributed. A group of core confounders, chosen *a priori* based on the current scientific literature (Costenbader et al. 2006; Liao et al. 2009; Uhlig et al. 1999) or study design, was included in the analyses and modeled as shown in Table 1 unless otherwise noted: age, non-Caucasian race, age at menarche, parity, total months of lactation, current menopausal status, menopausal hormone use, oral contraceptive use, physical activity, body mass index (BMI; modeled categorically), cigarette pack-years (calculated as the number of packs per day multiplied by the number of years of cigarette smoking), current smoking status, and socioeconomic status measured by nurses’ educational level, occupation of both parents, marital status, and husband’s education (if applicable). These variables were obtained from the questionnaire cycle that corresponded to the addresses used in the 1988 analysis (i.e., we used nurses’ BMI reported in the 1988 questionnaire for the analysis of 1988 addresses, and the BMI reported in the questionnaire at year of diagnosis or censoring for the analysis of addresses at time of diagnosis or censoring).

We used a loess smooth, which adapts to changes in population density (Hastie and Tibshirani 1990) and determined the optimal amount of data for the smooth, or span size, for each map by minimizing the Akaike’s Information Criterion (AIC). A rectangular grid covering the continental United States was created using the minimum and maximum longitude and latitude from the study subjects. Because GAMs may exhibit biased behavior at the edges of the data, the study area for spatial model predictions is the continental United States, excluding regions of low population density along the geographic edges of our study population. Using sensitivity analyses to determine the impact of sparse data areas on the predicted results, we identified the midwest (Great Plains), northern Maine, and southwest Texas as low population density regions. In these regions, a participant’s nearest neighbor was > 200 km away along the geographic edges of the study population. We converted log odds to odds ratios (ORs) using the odds of disease in the whole study area as the reference.

GAMs also provide a framework for hypothesis testing. We first tested the null hypothesis that case status does not depend on the smooth term, using a permutation test based on the difference of the deviances of equation 1with and without the smooth term (Webster et al. 2006). We discuss results as significant if the associated *p*-values are < 0.05, but acknowledge that some results may be due to chance. If the global deviance test indicated that geographic location is important, we examined pointwise departures from the null hypothesis using permutation tests to identify areas of the map that exhibit unusually high or low disease odds. We used contour lines to denote areas of significant decreased RA risk (points that ranked in the lower 2.5% of the pointwise permutation distributions, indicating low disease odds) or increased RA risk (upper 2.5% of the pointwise permutation distributions, indicating high disease odds). We used S-Plus (version 8.0; Insightful Corp., Somerville, MA) to perform the generalized additive modeling and a geographic information system (ArcGIS, version 9.3; ESRI, Redlands, CA) to map the results of our analyses. The institutional review boards of Boston University Medical Center and Brigham and Women’s Hospital approved the research.

## Results

Participants were predominantly white and > 50 years old (Table 1). Cases were more likely to be former smokers and current users of postmenopausal hormones. Higher proportions of controls were never-smokers and breast fed for at least 1 year. As expected, more women were postmenopausal at time of diagnosis (for cases) or censoring (for controls) than in 1988. There were also more physically active women and fewer current smokers at the time of diagnosis or censoring. Cases were more likely to have moved than were controls; 31.4% (145/461) of cases moved between 1988 and year of diagnosis, whereas 26.6% (2,455/9,220) of controls moved between 1988 and year of censoring. Covariate data were missing for < 10% of participants with the exception of information on postmenopausal hormone use (17%) and physical activity (23%).

**Figure 1:**
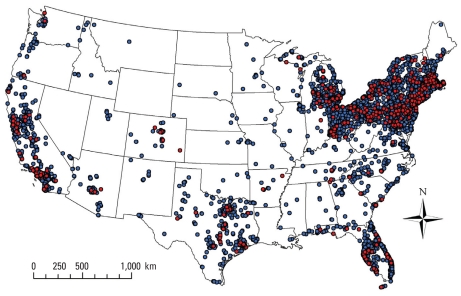
Distribution of cases and controls for RA. Each point represents the residences for cases (red) at diagnosis and controls (blue) at time of censoring. Locations have been geographically altered to preserve confidentiality.

Figure 1 shows the distribution of RA cases and controls in the United States using address at diagnosis or censoring. To preserve confidentiality, the figure was created by randomly placing residences within a small grid that included the actual location. Actual locations were used in the analysis.

**Figure 2:**
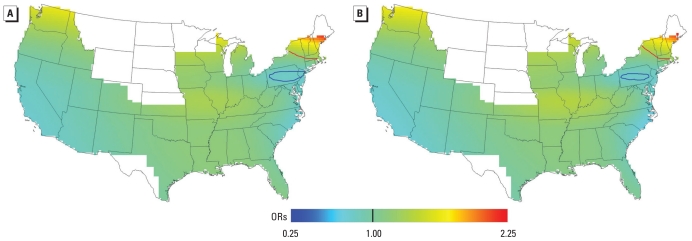
Results for addresses at time of diagnosis or censoring. ORs are relative to the whole study area. (*A*) Crude, optimal span of 0.55 (global *p* = 0.02). (*B*) Adjusted, optimal span of 0.50 (global *p* = 0.034). Contour lines denote areas of significantly increased (red) and decreased (blue) risk at the 0.05 level. Geographic patterns are similar for crude and adjusted analyses.

When geographic variation in RA risk was examined using addresses at diagnosis or censoring, the crude and adjusted analyses (Figure 2A and 2B, respectively) predicted similar results. Because of low data density and thus unreliable estimates, we did not predict odds of RA for regions shown in white. The association between location and RA was statistically significant for both analyses (crude, global *p*-values = 0.02; adjusted, global *p*-value = 0.034), indicating that ORs of RA varied with geographic location. Contour lines denote areas where RA risk relative to the whole study area was significantly increased (red) and decreased (blue) at the 0.05 level. A statistically significant area of increased risk was identified in the upper northeast including Vermont, New Hampshire, and southern Maine. A significant area of decreased risk was located in Pennsylvania. The optimal spans for the crude and adjusted analyses were 0.55 and 0.5, respectively. Crude ORs (CORs) ranged from 0.76 to 2.26, only slightly larger than adjusted ORs (AORs), which ranged from 0.68 to 2.17.

**Figure 3:**
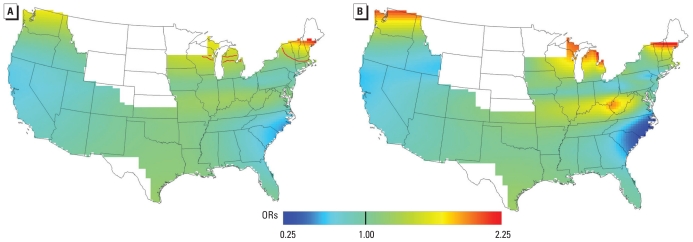
Results for addresses in 1988. ORs are relative to the whole study area. (*A*) Adjusted, optimal span of 0.55 (global *p* = 0.034); contour lines denote areas of significantly increased (red) and decreased (blue) risk at the 0.05 level. (*B*) Adjusted, span of 0.20. Small span size results in more spatial variation in risk.

Figure 3A shows the results of the adjusted analysis using 1988 residences with the optimal span of 0.55. Again, similar spatial patterns of predicted risk were found between the adjusted and crude analysis (not shown). Crude and adjusted maps predicted comparable ranges in ORs relative to the whole study area (COR = 0.61–2.39; AOR = 0.63–2.37), and both were statistically significant (global *p*-values of 0.029 and 0.034, respectively). We performed pointwise tests of significance and identified areas of higher risk in the northern areas in the midwest and northeast denoted by red contour lines. The AIC curve for the adjusted RA model indicated a local minima at span sizes of 0.20 before reaching the global minimum (and optimal span) of 0.55. We repeated the adjusted analysis using a span of 0.20 (Figure 3B). The small span of 0.20 produced a surface with more spatial variation in risk, including an area of high ORs along the Ohio River near West Virginia and northern Kentucky. The model also predicted even higher ORs in the northern latitudes, the west, the midwest (Great Plains), and the northeast. We did not test for statistical significance of location in this model because the optimal span size was not used.

## Discussion

Results of the spatial analysis are consistent with an earlier regional study conducted by Costenbader et al. (2008a) that found increased risk of RA for those women who lived in the midwest and northeast United States, compared with west of the Rocky Mountain range, and the association was stronger with residency at age 15 and 30 years than at baseline in 1976. They also observed elevated risk in the mid-Atlantic region compared with the area west of the Rocky Mountain range, which the current spatial analysis did not observe. Although both studies used the NHS data set, possible reasons for the difference in results include study population (the earlier study included women diagnosed with RA beginning in 1976 compared with 1988 in the current study), reference group (west of the Rocky Mountain range compared with the entire study area), and geographic scale (regional versus individual-level analyses). The time periods of the addresses were different as well. We examined risk of RA using addresses from 1988 (when the mean age for the current study population was 54 years old) and those at diagnosis or censoring. These two time points were not considered in the earlier regional study (Costenbader et al. 2008a). Although the NHS began in 1976, addresses were only geocoded beginning in 1988, which limited our ability to perform extensive space–time analyses (Vieira et al. 2008).

Spatial patterns were similar for addresses in 1988 and at the time of diagnosis or censoring (Figures 2B and 3A), although slightly higher ORs were observed for the 1988 analysis. This finding suggests that long-term exposure may be more important than recent exposure. We observed even higher ORs when we restricted the 1988 analysis to women who were diagnosed or censored at least 8 years later (1996 or later; data not shown). Although this restricted analysis was limited by small case numbers (*n* = 227), it supports our hypothesis that earlier rather than recent exposure may be more important. Regardless of timing, a statistically significant area in the upper northeast that included Vermont, New Hampshire, and southern Maine was identified as having consistently elevated RA risk relative to the whole study area, and an additional analysis (Figure 3B) predicted increased ORs for the more northern latitudes of the United States. A geographic association with northern latitudes has also been observed for multiple sclerosis and Crohn’s disease. These autoimmune diseases may be mediated by a reduction in vitamin D through decreased solar exposure and the immune effects of vitamin D deficiency (Armitage et al. 2004; Arnson et al. 2007; Hernán et al. 1999; Kamen et al. 2006; McLeod et al. 1994; Munger et al. 2006; Patel et al. 2007; Ponsonby et al. 2005; Sioka et al. 2009). The studies of dietary intake of vitamin D and incident RA have come to contradictory conclusions. Merlino et al. (2004) found a strong protective effect of high vitamin D intake in diminishing incident RA, whereas a study by Costenbader et al. (2008b) revealed no association between intake and incident RA. However neither study assessed vitamin D from solar exposure.

Geographic variation may also be due to other environmental exposures or residual spatial confounding. Spatial confounding occurs when risk factors for a disease are not evenly distributed. For example, a cluster of lung cancer may be due to an increased density of smokers. Crude and adjusted analyses produced similar geographic patterns of RA risk, and missing covariate data were not a concern in our analyses. Although we adjusted for individual-level socioeconomic status, some authors argue for the inclusion of group-level contextual variables (e.g., Krieger et al. 2002). By linking residential location to census data, one could test the importance of these variables relative to individual-level covariates. We are currently working on methods involving generalized additive mixed models to incorporate a smooth of location into a multilevel model adjusted for individual- and community-level risk factors. Our findings also may be due to geographic differences in the location of rheumatology specialists or in diagnosing practices.

These spatial analyses have some potential limitations. GAMs may exhibit biased behavior at the edges of the data, although our work with synthetic data suggested little to no bias when a loess smooth is used (Webster et al. 2006). To reduce the likelihood of bias from edge effects, we did not predict ORs in regions of low data density, which restricted the extent of northern latitudes available for our analysis. We used the AIC to choose an optimal span, but when we used a smaller span of 0.20 in our analyses, we were able to discern greater spatial variation that may be of importance. Although there is some benefit to having a non–*ad hoc* method for span selection, analyses should not be limited to just one span. In the current analyses, we identified areas with significantly increased or decreased risk using pointwise hypothesis tests only if global tests were statistically significant, but performing multiple testing at each location may result in an increase in the type I error rate. In addition, many epidemiologists prefer confidence intervals when evaluating the precision of point estimates in addition to *p*-values (Rothman and Greenland 1998). It should be possible to compute variance bands (also known as confidence bands) for our maps, but displaying three surfaces of ORs makes it difficult to visually interpret points where the bands do not include one (Hastie and Tibshirani 1990).

Prospective cohort studies are one of the standard epidemiologic tools for investigating associations between disease and exposure. By combining such data with advanced statistical techniques, we were able to address many criticisms of spatial studies. Self-reported cases were confirmed by examining medical records, and controls selected from among the noncases provided an estimate of the underlying, nonuniform population from which the cases arose. Because the data are from a prospective cohort, selection bias is not a concern in this study. However, results for this spatial analysis of a female nurse cohort may not be generalizable to other populations. Point-based data were used, avoiding aggregation within administrative boundaries. We were able to control for a large number of covariates, which can be done only to a limited degree using other cluster analysis tools like the scan statistic (Kulldorff 1997). Residential history information allowed us to take calendar year into account, potentially quite important for diseases with environmental risk factors. Although spatial analyses are useful for generating new hypotheses or supporting existing hypotheses, areas of increased and decreased ORs should be considered exploratory. Further analysis that examines the relationship between vitamin D exposure and RA is warranted to explore these results.

## Conclusions

Using GAMs and GIS, we generated maps of RA risk relative to the whole study area. When available, prospective cohort studies provide extensive data on potential risk factors and residential histories that address many methodological criticisms of cluster studies. We identified a significant area of increased ORs in the northeast, and additional analyses suggest that women living in more northern latitudes may be at greater risk for RA relative to the whole study area. Similar geographic associations have been observed with other chronic autoimmune diseases including multiple sclerosis. The results of the current analysis illustrate the application of GAMs and GIS to visualize geographic variation in RA risk, adjust for known confounders, and test for the statistical significance of location. Our method is particularly useful in generating hypotheses for further investigation and supporting existing hypotheses, especially when residential histories are available.

## Figures and Tables

**Table 1:** Selected characteristics of cases and controls in 1988 and at the time of diagnosis or censoring.

	Cases (*n* = 461)		Controls (*n* = 9,220)	
Characteristics	1988	Dx	1988	Dx
Mean age (years)^a^	54.8	61.4	54.4	61.0
BMI (kg/m^2^)	23.5	26.0	22.5	24.5
Mean age at menarche (years)	12.4	12.4	12.4	12.4
Pack-years of smoking (mean)^b^	26.3	27.8	23.0	24.6
Caucasian race (%)	94.1	94.1	93.8	93.8
Smoking status (%)				
Current	18.4	13.2	19.1	13.8
Former	40.8	48.6	34.2	40.0
Never	37.7	37.7	43.8	43.6
Parity/lactation (%)				
Nulliparous	6.7	6.7	7.0	7.0
Parous, never breast-fed	34.1	34.1	29.5	29.5
Parous, breast-fed 1–11 months	36.9	36.9	35.0	35.0
Parous, breast-fed ≥ 12 months	12.8	12.8	15.8	15.8
Menopausal status (%)				
Premenopausal	20.8	6.3	25.8	10.7
Postmenopausal	75.3	93.1	68.7	85.9
Unknown status	3.9	0.6	5.5	3.4
Postmenopausal hormone use (%)^c^				
Never used	50.7	33.0	52.4	33.7
Past use	13.4	19.7	11.7	17.9
Current use	26.2	40.6	19.3	31.8
Oral contraceptive use (%)				
Never used	48.6	48.6	50.2	50.2
Ever used	48.6	48.6	45.0	45.0
Physical activity (metabolic equivalent hours/week, %)				
< 3	17.6	20.8	17.0	17.2
3 to < 9	23.6	20.0	19.7	18.1
9 to < 18	16.9	20.2	15.6	16.0
18 to < 27	10.2	10.8	8.7	9.9
≥ 27	15.2	19.3	12.9	15.9
Father’s occupation (%)				
Professional/manager	23.6	23.6	25.4	25.4
Other job	76.4	76.4	74.6	74.6
Mother’s occupation (%)				
Housewife	67.5	67.5	64.2	64.2
Other job	32.5	32.5	35.8	35.8
Education (%)				
Nurse	84.2	84.2	72.4	72.4
Other	15.8	15.8	27.6	27.6
Marital status (%)				
Married	71.6	71.6	63.2	63.2
Other	28.4	28.4	36.8	36.8
Husband’s education (%)				
Missing or not applicable	22.8	22.8	34.6	34.6
< High school	6.1	6.1	3.9	3.9
High school	31.7	31.7	25.7	25.7
> High school	39.5	39.5	35.7	35.7

Dx, diagnosis or censoring.

a Age was originally modeled in months but converted to years for this paper.

b Among ever-smokers.

c Among postmenopausal women.
